# Supplementary figures and images for: Identification of Transcription Factor Networks during Mouse Hindlimb Development
Source: Cells. 2022 Dec 21;12(1):28. doi: 10.3390/cells12010028 (PMC9818828; doi:10.3390/cells12010028)

A

original

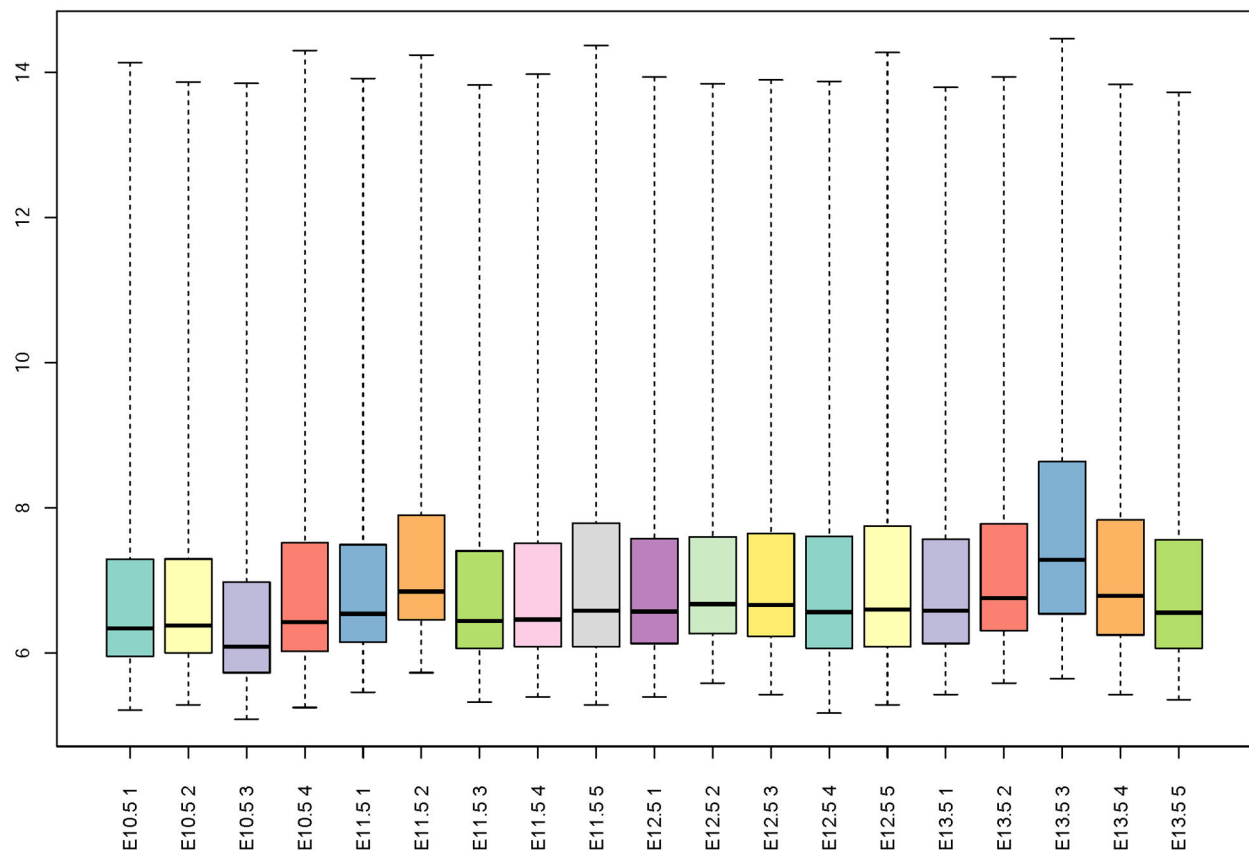

B

RMA

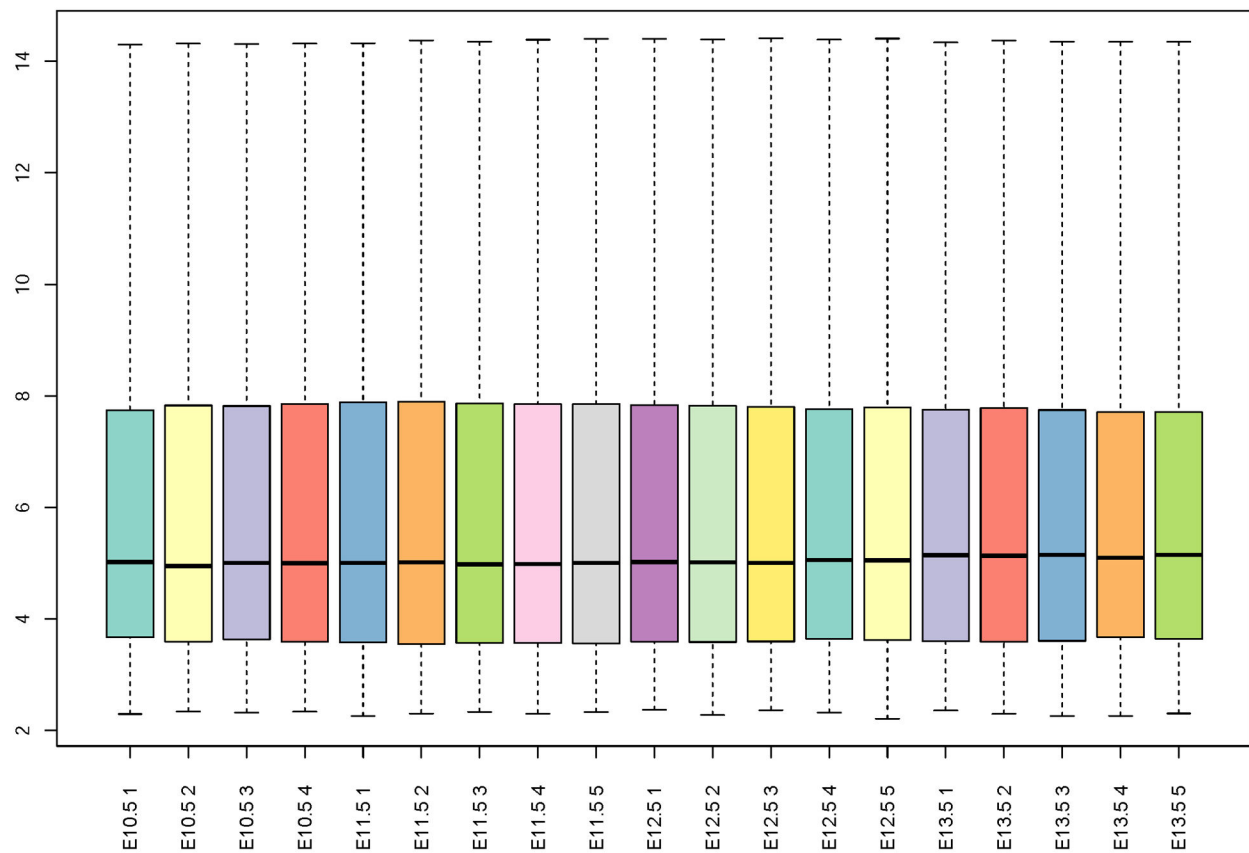

Supplement: Supplementary file 1 [file cells-12-00028-s001.zip › Figure S1.pdf]

E10.5

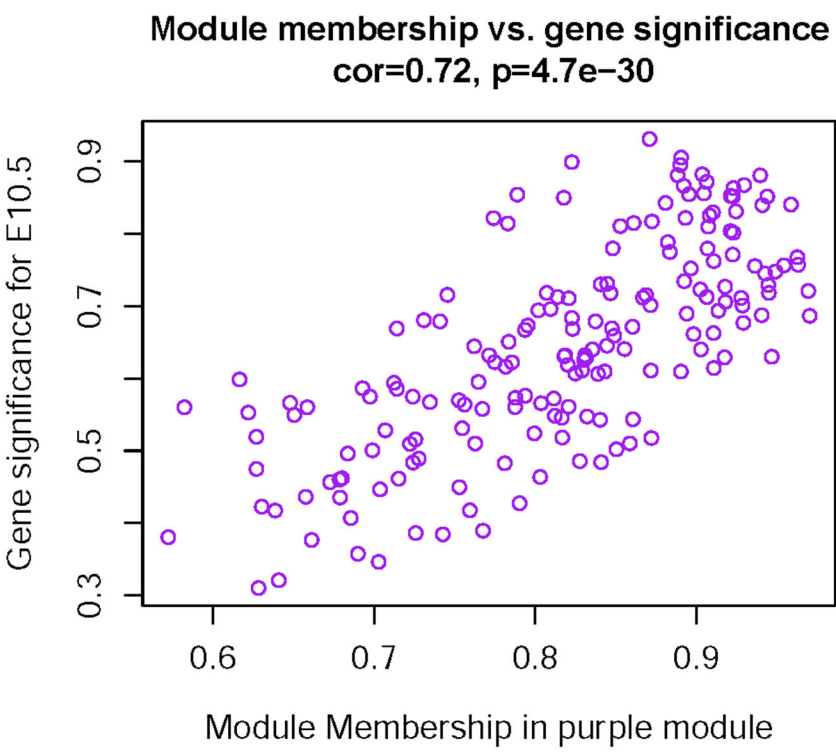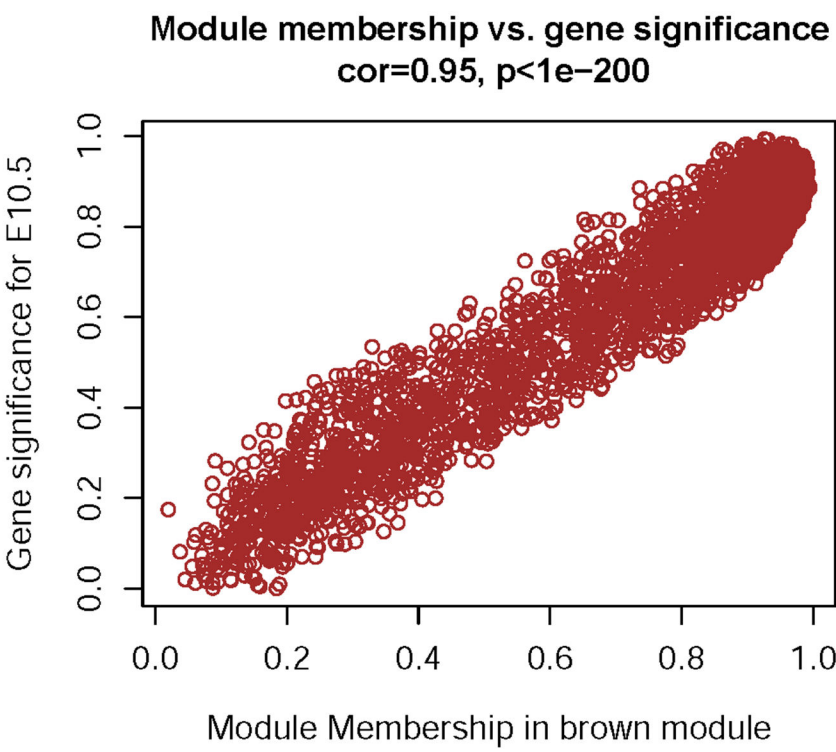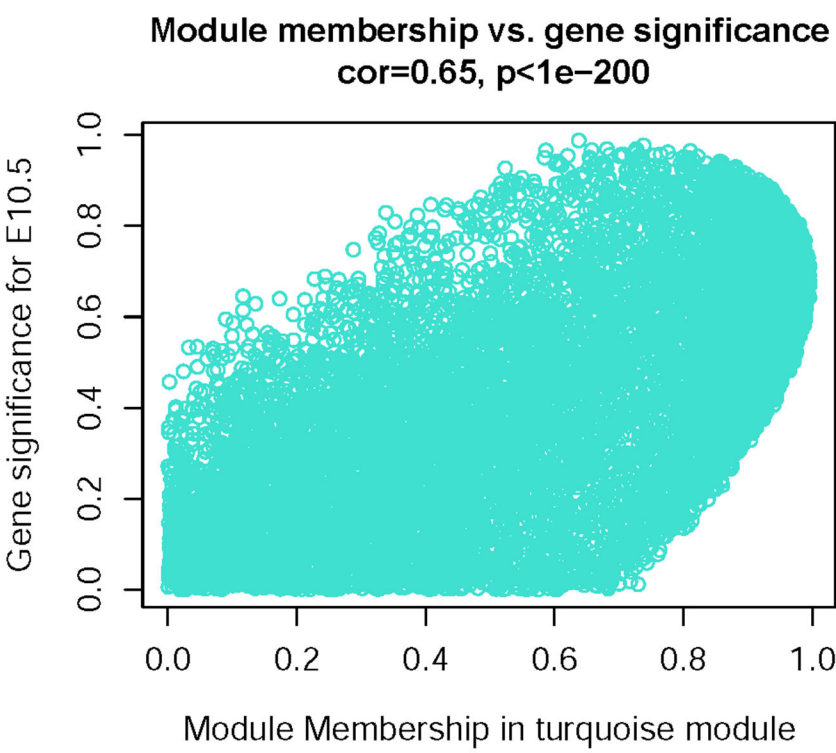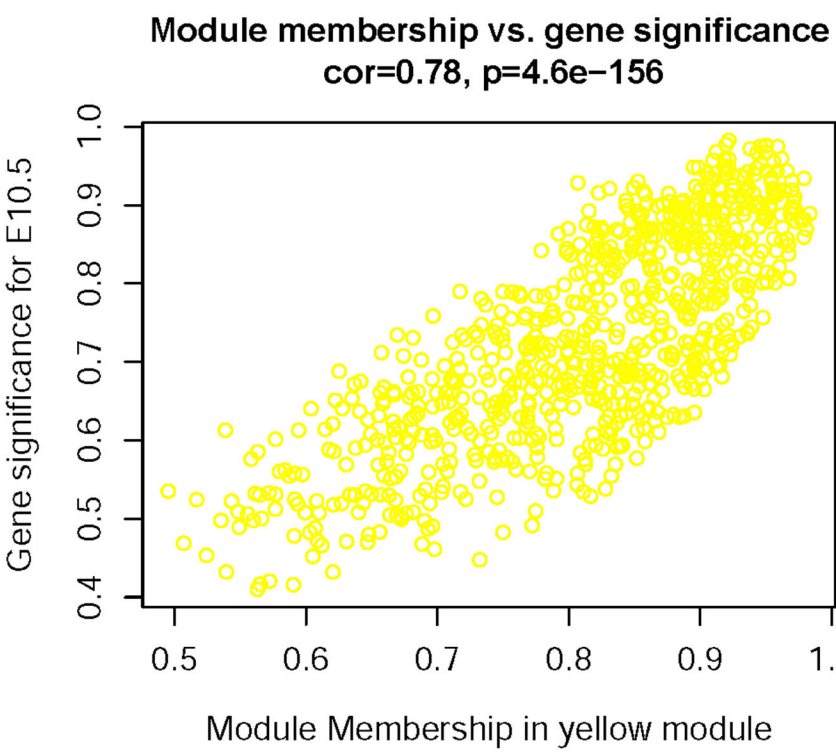

E13.5

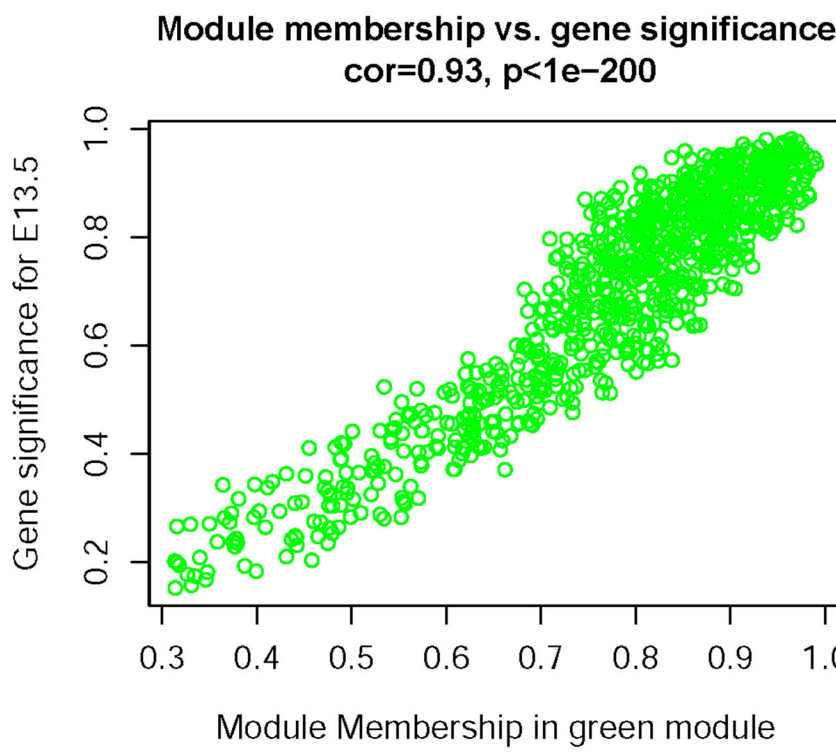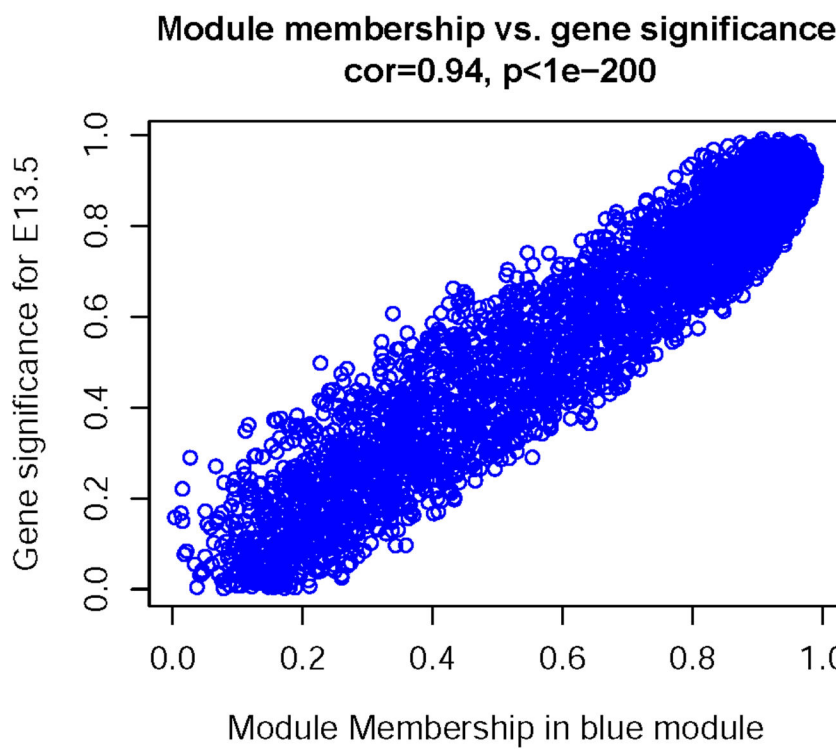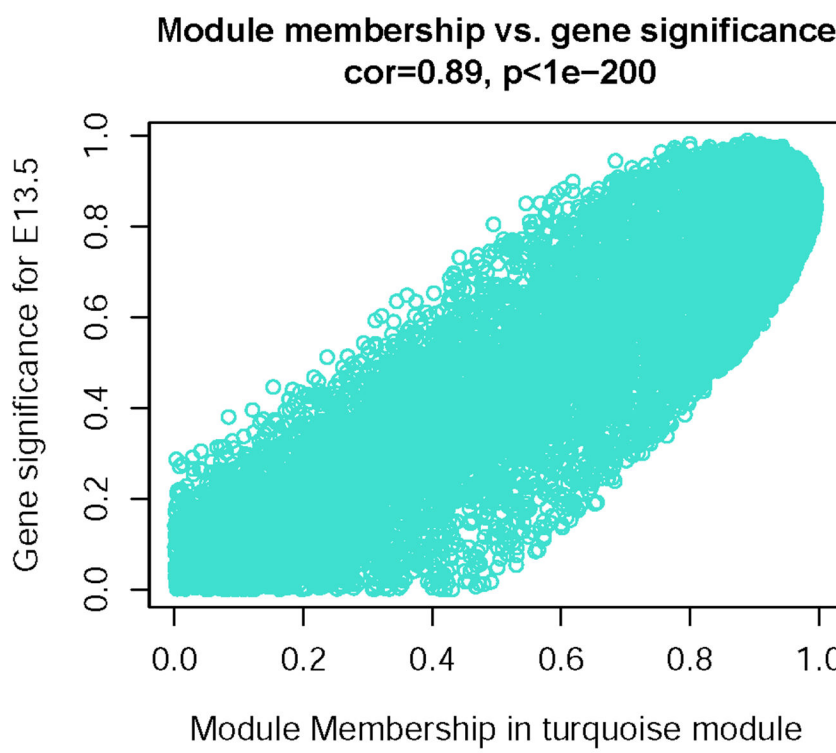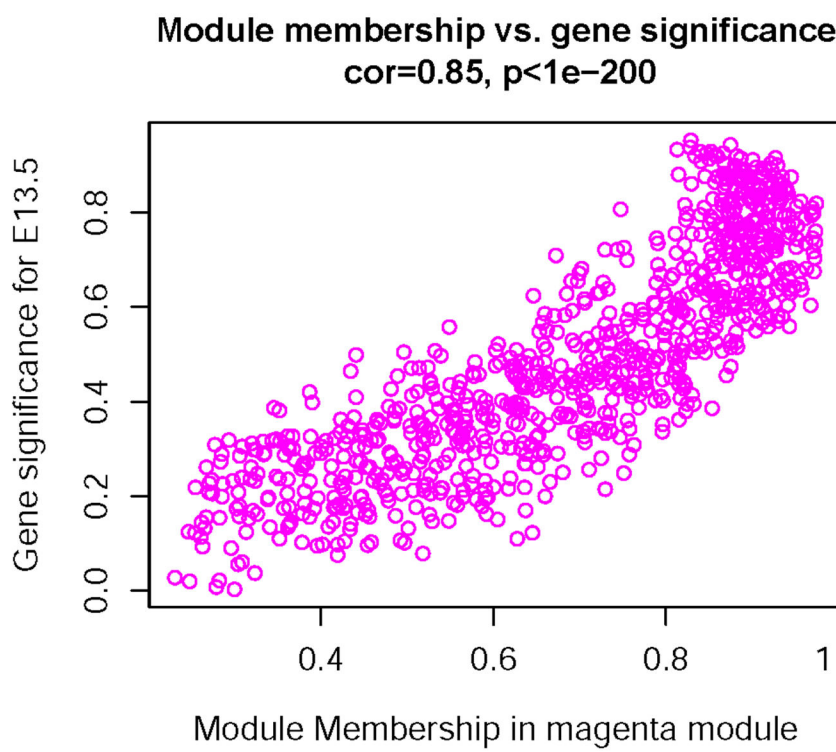

Supplement: Supplementary file 1 [file cells-12-00028-s001.zip › Figure S2.pdf]

A

Microarray

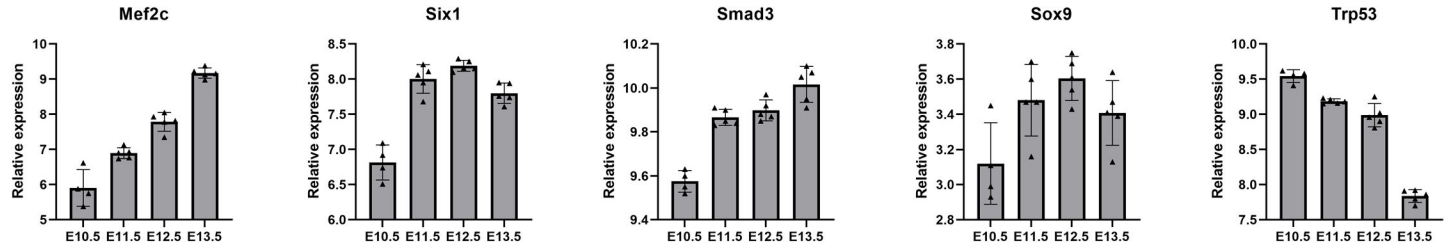

qRT-PCR

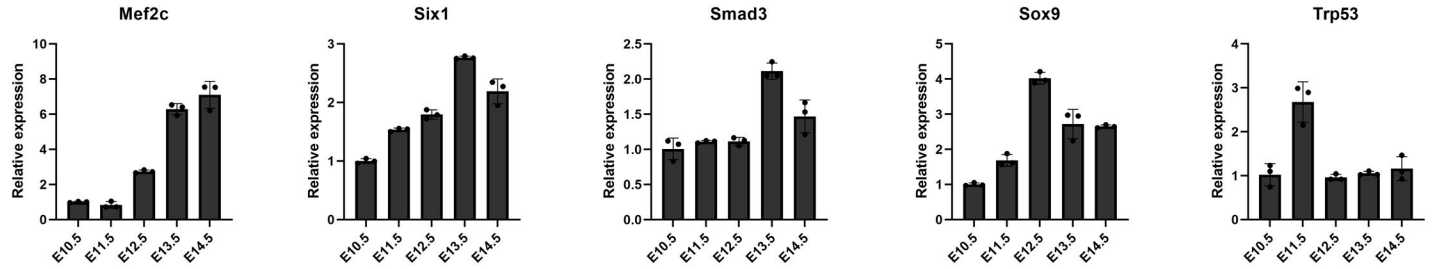

B

Eigengene adjacency heatmap

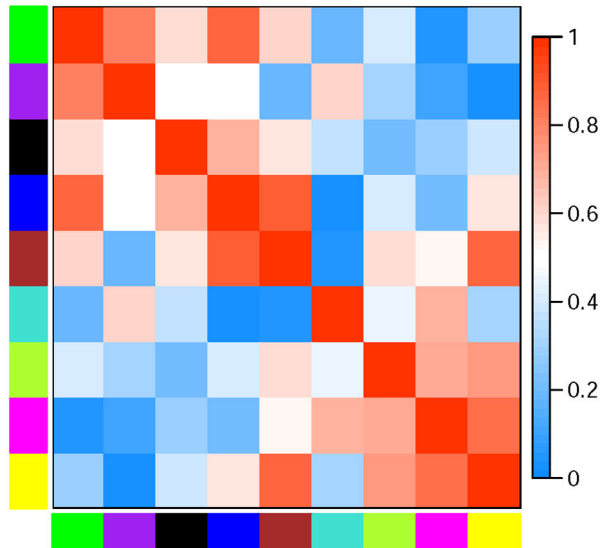

C

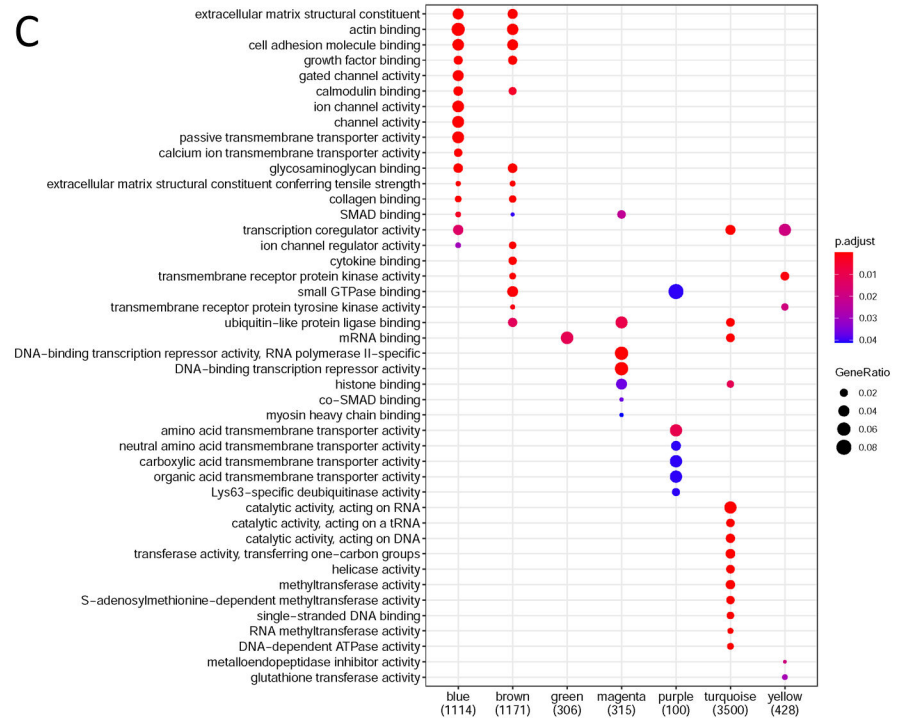

Supplement: Supplementary file 1 [file cells-12-00028-s001.zip › Figure S3.pdf]

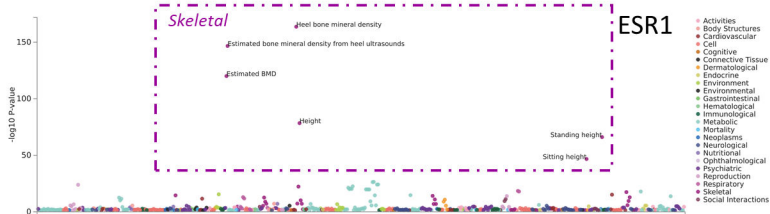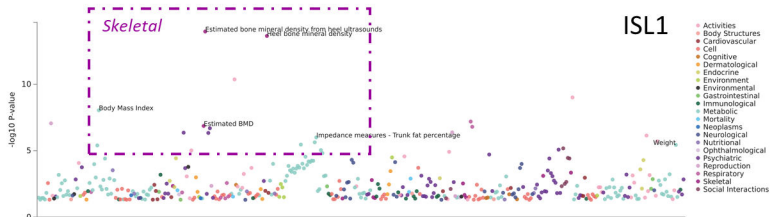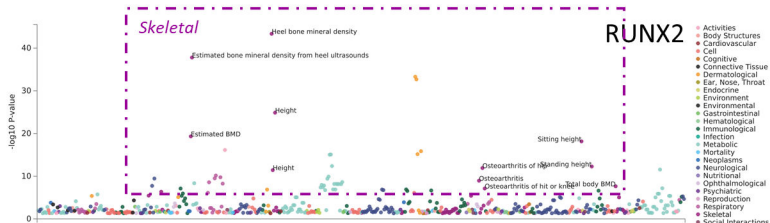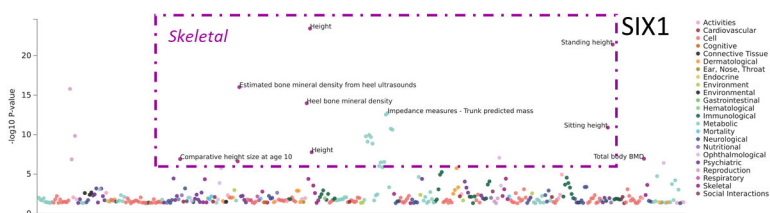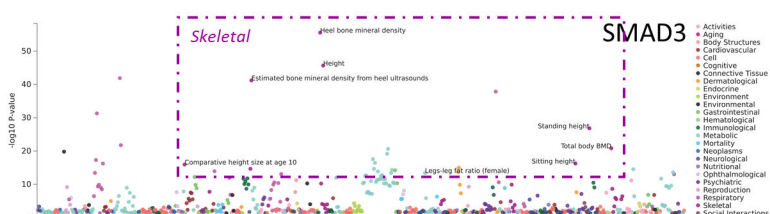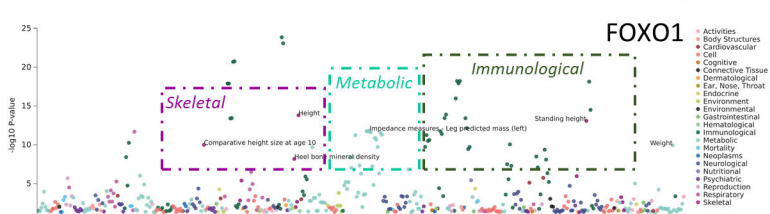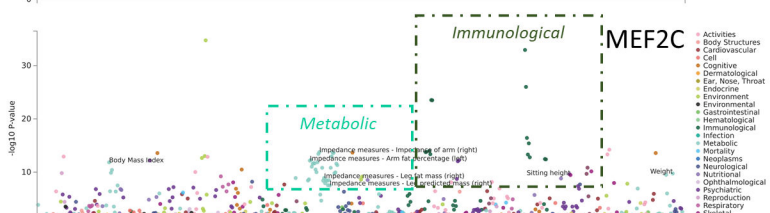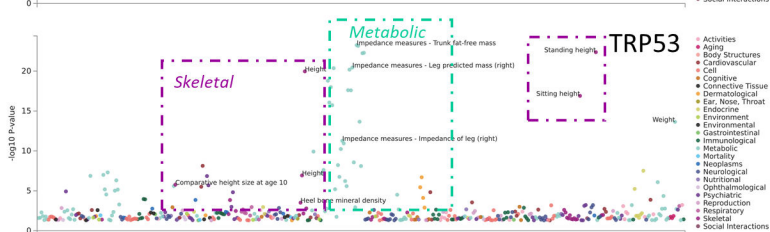

Supplement: Supplementary file 1 [file cells-12-00028-s001.zip › Figure S4.pdf]
